# When Clinical Suspicion Outweighs a Negative Biopsy: Early Tongue Squamous Cell Carcinoma Diagnosed After Persistent Oral Leukoplakia

**DOI:** 10.7759/cureus.114040

**Published:** 2026-08-06

**Authors:** Pedro Reboredo, Paula Nogueira, Nina Boer, Fátima Cereja, Inês Barbosa, Cristina Sousa

**Affiliations:** 1 Internal Medicine, Unidade Local de Saúde do Algarve - Hospital de Faro, Faro, PRT

**Keywords:** case report, clinicopathological discordance, diagnostic persistence, negative biopsy, oral leukoplakia, tongue squamous cell carcinoma

## Abstract

Oral leukoplakia is the most common oral potentially malignant disorder and represents a well-recognized precursor of oral squamous cell carcinoma (OSCC). Although incisional biopsy remains the diagnostic gold standard, false-negative results may occur because of sampling error and lesion heterogeneity, creating a potential discordance between histopathological findings and clinical evolution. We report the case of a 67-year-old former heavy smoker who presented with a two-year history of persistent leukoplakia involving the right lateral border of the tongue, accompanied by an unintentional 20-kg weight loss over the same period. An initial incisional biopsy demonstrated no epithelial dysplasia, and the lesion was attributed to chronic mechanical irritation related to a dental implant. Despite adjustment of the dental prosthesis, the lesion remained unchanged, prompting further diagnostic evaluation. Following exclusion of metabolic causes, continued clinical suspicion prompted referral to a tertiary head and neck oncology center, where partial glossectomy revealed a moderately differentiated invasive squamous cell carcinoma (pT1N0M0). The patient underwent curative surgical treatment and remained free of recurrence after one year of follow-up. This case highlights the importance of integrating histopathological findings with the patient's evolving clinical picture and demonstrates that persistent clinicopathological discordance should prompt diagnostic reassessment. Ultimately, a negative biopsy should not represent the endpoint of investigation when clinical suspicion remains high.

## Introduction

Oral leukoplakia is the most common oral potentially malignant disorder and represents one of the principal precursor lesions of oral squamous cell carcinoma (OSCC) [1,2]. Although the overall risk of malignant transformation varies considerably according to clinical, histopathological, and patient-related factors, lesions involving the lateral border of the tongue and occurring in individuals with significant tobacco exposure are associated with a substantially higher risk of malignancy [1,2,3].

Histopathological examination following incisional biopsy remains the diagnostic gold standard for suspicious oral lesions and is fundamental for guiding clinical management [4,5]. However, biopsy is not without limitations. Sampling error, lesion heterogeneity, and the multifocal distribution of epithelial dysplasia may result in false-negative findings when the sampled tissue is not representative of the lesion as a whole [1,4]. Consequently, histopathological findings should always be interpreted in conjunction with the patient's clinical presentation and subsequent evolution.

A discrepancy between persistent clinical suspicion and apparently reassuring histopathological findings, referred to as clinicopathological discordance, should prompt diagnostic reassessment rather than diagnostic closure. Current recommendations emphasize that persistent or progressive oral lesions require repeat biopsy, excisional biopsy, or referral to a specialist center when the clinical course remains inconsistent with the initial diagnosis [4,5,6].

We report the case of a patient with persistent oral leukoplakia in whom an initial benign biopsy delayed the definitive diagnosis of early tongue squamous cell carcinoma. This case highlights the importance of diagnostic persistence and careful clinicopathological correlation when evaluating persistent oral lesions with apparently reassuring histopathological findings.

## Case presentation

A 67-year-old man with metabolic syndrome, under regular follow-up at the Internal Medicine outpatient clinic, reported a persistent leukoplakic lesion involving the right lateral border of the tongue during a routine consultation. The lesion had first appeared approximately two years earlier, shortly after placement of a dental implant, and had remained persistent throughout this period. The patient also described an unintentional weight loss of approximately 20 kg over the same two-year interval. His medical history included well-controlled hypertension, type 2 diabetes mellitus, dyslipidemia, and benign prostatic hyperplasia. He was a former heavy smoker (84 pack-years), having quit smoking nine years earlier, and had a history of regular alcohol consumption, which he had subsequently discontinued.

**Figure 1:**
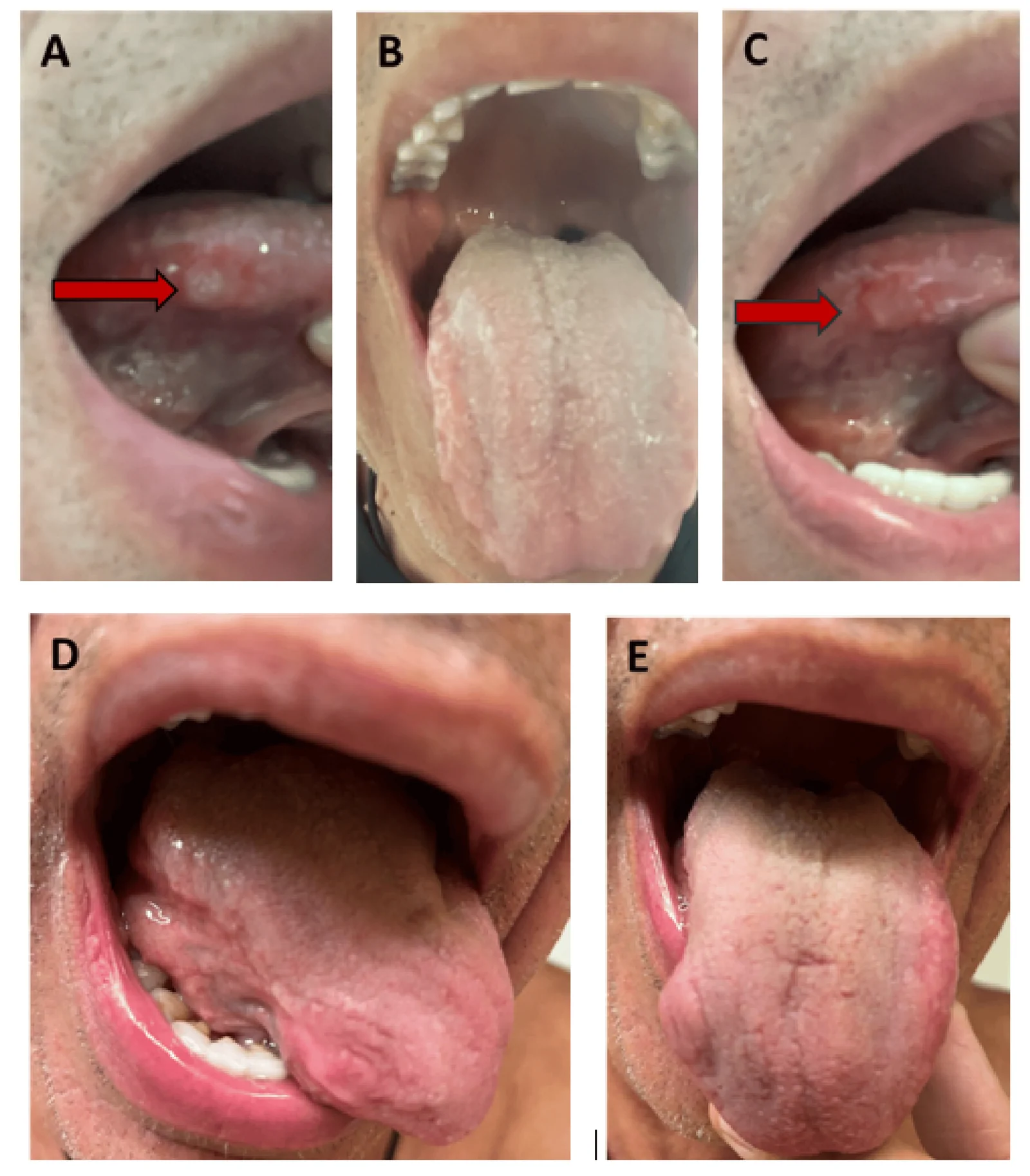
Clinical appearance of the tongue lesion before and after surgical treatment. (A–C) Preoperative clinical photographs demonstrating the persistent leukoplakic lesion involving the right lateral border of the tongue before the initial incisional biopsy. (D-E) Postoperative appearance following partial glossectomy, showing satisfactory healing without clinical evidence of residual disease.

Given the patient's progressive weight loss, a comprehensive Internal Medicine evaluation was undertaken, excluding metabolic and other common systemic causes. Considering the persistence of the oral lesion over a two-year period, the patient was referred to the Department of Otorhinolaryngology at Hospital de Faro, where he was first evaluated in September 2024. Clinical examination revealed a persistent leukoplakic lesion with focal mucosal erosion involving the right lateral border of the tongue (Figure 1A-1C). An incisional biopsy was performed, and histopathological examination demonstrated hyperplastic stratified squamous epithelium with focal erosion/ulceration and no evidence of epithelial dysplasia in the sampled tissue. Cervical computed tomography showed no significant abnormalities. Considering the temporal relationship between lesion onset and dental implant placement, together with the benign histopathological findings and the absence of suspicious imaging features, the lesion was considered most consistent with chronic mechanical irritation, and adjustment of the dental prosthesis was recommended.

Following adjustment of the dental prosthesis, the lesion remained clinically unchanged despite elimination of the presumed traumatic stimulus. Given the persistence of the lesion, the patient's heavy smoking history, ongoing constitutional symptoms, and the lack of an alternative explanation for his weight loss, the initial diagnosis no longer fully accounted for the evolving clinical picture. Consequently, the patient was referred to the Department of Otorhinolaryngology at the Portuguese Institute of Oncology (IPO), Lisbon, for further evaluation.

**Figure 2:**
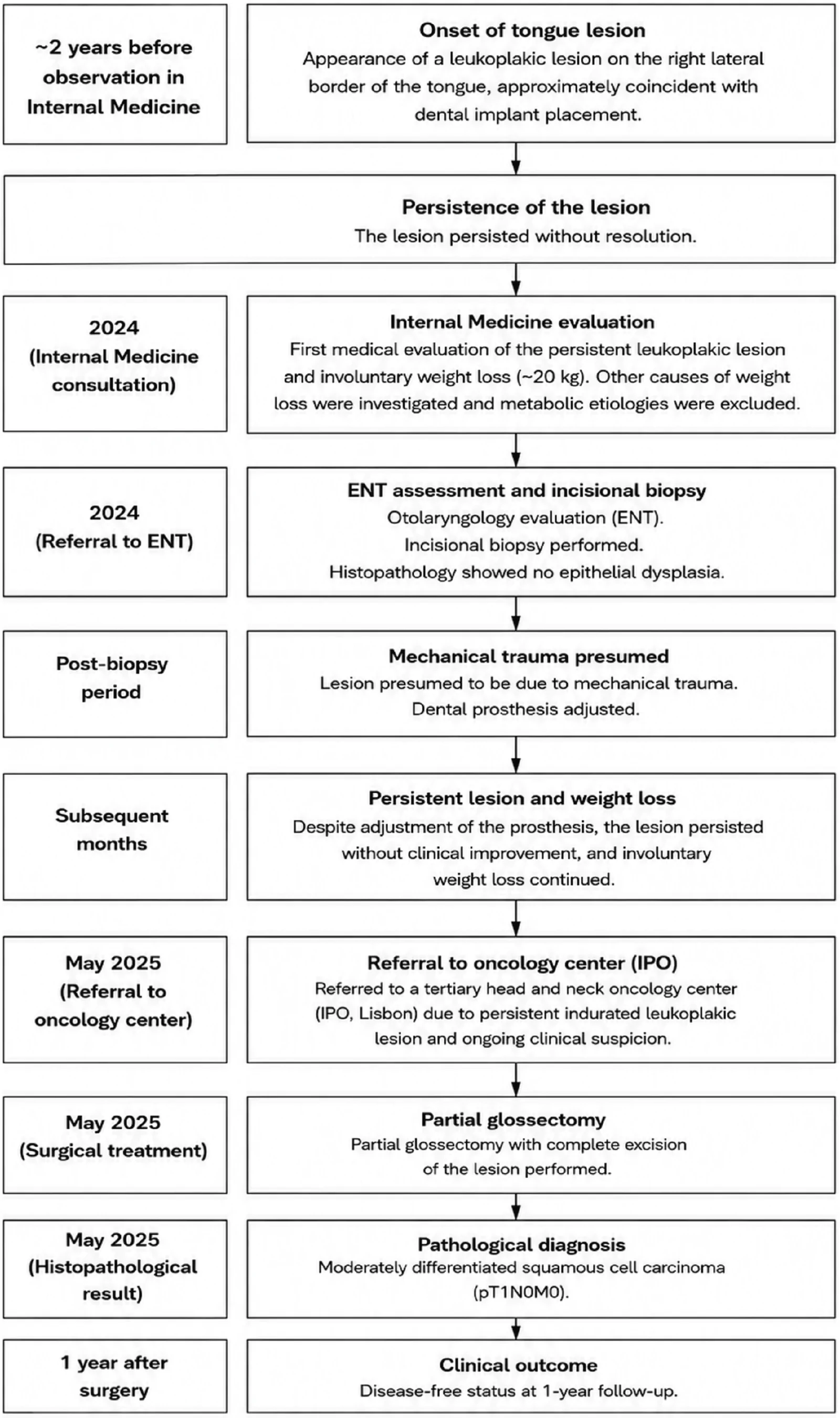
Diagnostic pathway illustrating the persistence of clinical suspicion despite an initially benign biopsy. Flowchart summarizing the patient's diagnostic pathway from the onset of a persistent leukoplakic lesion to the diagnosis of early-stage tongue squamous cell carcinoma and one-year disease-free follow-up. The figure highlights the clinicopathological discordance between the initial benign incisional biopsy and the subsequent clinical evolution that prompted referral to a tertiary head and neck oncology center and definitive surgical treatment. Abbreviations: ENT, otorhinolaryngology; IPO, Portuguese Institute of Oncology of Lisbon

The patient's diagnostic pathway is summarized in Figure 2.

At the initial IPO consultation in May 2025, physical examination revealed a firm leukoplakic lesion measuring approximately 1 × 1 cm on the right lateral border of the tongue, without palpable cervical lymphadenopathy or other abnormalities of the upper aerodigestive tract. Given the persistent clinical suspicion, the patient underwent partial glossectomy with complete macroscopic excision later that month. Histopathological examination revealed a moderately differentiated invasive squamous cell carcinoma measuring 1.6 cm in greatest dimension, with a depth of invasion of 4 mm, no lymphovascular or perineural invasion, and clear surgical margins. The final pathological stage was pT1N0M0.

Postoperative cervical ultrasonography demonstrated no pathological cervical lymphadenopathy, while magnetic resonance imaging of the head and neck showed no evidence of locoregional disease. The patient subsequently underwent regular clinical and radiological surveillance and remained free of local, regional, or distant recurrence after one year of follow-up. The postoperative appearance of the surgical site demonstrated satisfactory healing, with no evidence of residual or recurrent disease on clinical examination (Figure 1D, 1E).

This case illustrates how persistent clinical suspicion, despite an initially benign biopsy, enabled diagnosis at an early stage and ultimately allowed curative surgical treatment.

## Discussion

Oral leukoplakia is the most common oral potentially malignant disorder and remains one of the principal precursor lesions of OSCC. Although the reported risk of malignant transformation varies considerably according to clinical and histopathological characteristics, lesions involving the lateral border of the tongue, particularly in patients with significant tobacco exposure, warrant careful evaluation and long-term surveillance [1-3]. Incisional biopsy remains the diagnostic gold standard; however, clinicians should recognize that a single benign histopathological result does not completely exclude malignancy when clinical suspicion persists [4,5].

One of the major diagnostic challenges in oral potentially malignant disorders is the possibility of false-negative biopsy results. This limitation is primarily related to sampling error, lesion heterogeneity, and the multifocal nature of epithelial dysplasia. Dysplastic or invasive areas may coexist with benign or hyperkeratotic epithelium within the same lesion, meaning that an incisional biopsy may fail to sample the most representative area [1,4]. Consequently, histopathological findings should always be interpreted within the broader clinical context rather than in isolation. This concept is reflected in our patient's initial pathology report, which appropriately stated that no epithelial dysplasia was identified "in the sampled tissue," acknowledging the inherent limitations of tissue sampling.

In the present case, the initial diagnostic approach was clinically justifiable. The lesion developed shortly after placement of a dental implant, cervical imaging was unremarkable, and the initial biopsy demonstrated no evidence of epithelial dysplasia. Furthermore, chronic mechanical irritation is a frequent cause of persistent oral ulceration and leukoplakic lesions encountered in everyday clinical practice. The purpose of this report is therefore not to criticize the initial management, but rather to emphasize that diagnostic hypotheses should be continuously reassessed as new clinical information becomes available.

Following adjustment of the dental prosthesis, the expected clinical improvement did not occur. The lesion remained clinically unchanged, while the previously documented involuntary weight loss remained unexplained despite an extensive investigation excluding metabolic causes. Combined with an 84 pack-year smoking history and persistent induration of a lesion located at one of the highest-risk anatomical sites for OSCC, these findings created a clear clinicopathological discordance. In such circumstances, continued reliance on the initial biopsy alone would inadequately explain the patient's evolving clinical picture. Rather than disregarding the histopathological findings, clinicians should recognize that pathology and clinical assessment are complementary components of the diagnostic process. When these diverge, repeat biopsy, excisional biopsy, or referral to a specialized center should be strongly considered [4-7].

This case also highlights the importance of longitudinal clinical assessment. The diagnosis was not established because of a single investigation, but because the patient's clinical evolution prompted reconsideration of the initial working diagnosis. From an Internal Medicine perspective, the presence of unexplained constitutional symptoms, together with a persistent focal lesion and significant oncological risk factors, justified maintaining a broad differential diagnosis despite apparently reassuring investigations. This integrated clinical reasoning ultimately led to referral to a tertiary head and neck oncology center, where partial glossectomy confirmed an early-stage moderately differentiated squamous cell carcinoma (pT1N0M0).

Early diagnosis remains the single most important prognostic factor in OSCC. Patients diagnosed with localized disease have significantly better survival outcomes and are frequently managed with surgery alone, avoiding multimodal treatment and its associated morbidity [1,8]. In our patient, persistent clinical suspicion despite an initially benign biopsy enabled definitive diagnosis while the tumor remained at an early pathological stage, allowing curative surgical treatment with no evidence of recurrence after one year of follow-up. As a single case report, these observations cannot establish the frequency of false-negative biopsy findings or be generalized to all patients with oral leukoplakia; rather, they illustrate the importance of continued clinical reassessment when clinicopathological discordance persists.

Ultimately, this case reinforces a fundamental principle of clinical medicine: diagnostic tests should support, rather than replace, clinical reasoning. A negative biopsy should never be interpreted as the endpoint of diagnostic evaluation when the patient's clinical course continues to suggest otherwise. Persistent clinicopathological discordance should prompt clinicians to reconsider the diagnosis, pursue further investigation and, when appropriate, seek multidisciplinary assessment. In this patient, diagnostic persistence transformed an initially reassuring investigation into the early diagnosis of a potentially life-threatening malignancy.

## Conclusions

This case illustrates that diagnostic evaluation should remain a dynamic process, particularly when the patient's clinical evolution is inconsistent with initially reassuring investigations. Persistent oral lesions in high-risk individuals require ongoing reassessment, even after a benign biopsy, if clinical suspicion remains high.

Rather than relying on histopathological findings in isolation, clinicians should integrate pathology with the patient's overall clinical presentation and disease course. In this case, persistent clinicopathological discordance prompted further investigation, leading to the diagnosis of an early-stage tongue squamous cell carcinoma and curative surgical treatment.

Ultimately, a negative biopsy should never mark the end of the diagnostic journey when the clinical picture continues to suggest otherwise.
